# Phylogenetic relationship and characterization of the complete mitochondrial genome of *Cheilomenes sexmaculata* (Coleoptera: Coccinellidae)

**DOI:** 10.1080/23802359.2021.1955767

**Published:** 2021-07-21

**Authors:** Gaoqi Cheng, Yimin Du, Xinjun Liu

**Affiliations:** a School of Life Sciences, Gannan Normal University, Ganzhou, China; b National Navel Orange Engineering and Technology Research Center, Ganzhou, China

**Keywords:** Ladybird, *Cheilomenes sexmaculata*, mitochondrial genome, phylogenetic analysis

## Abstract

*Cheilomenes sexmaculata* is a common natural enemy for aphid and psyllid in agricultural systems in South China. In this study, we sequenced and analyzed the complete mitochondrial genome (mitogenome) of *C. sexmaculata*. This mitogenome was 17,297 bp long and encoded 13 protein-coding genes (PCGs), 22 transfer RNA genes (tRNAs) and two ribosomal RNA unit genes (rRNAs). Gene order was conserved and identical to most other previously sequenced Coccinellidae. All PCGs of *C. sexmaculata* have the conventional start codon for invertebrate mitochondrial PCGs (ATN), with the exception of *cox1* (AAT) and *nad3* (TTG). Except for seven genes (*cox1*, *cox2*, *cox3*, *nad3*, *nad5*, *nad4* and *nad6*) end with the incomplete stop codon T−, all other PCGs terminated with the stop codon TAA or TAG. The whole mitogenome exhibited heavy AT nucleotide bias (78.0%). Phylogenetic analysis positioned *C. sexmaculata* in a well-supported clade with *Aiolocaria hexaspilota*. The relationships (Sticholotidinae + (Coccinellinae + (Scymninae + Epilachninae))) were supported in Coccinellidae, and Halyziini was paraphyletic to Coccinellini within Coccinellinae.

Coccinellidae is commonly called Ladybirds which belong to the superfamily Cucujoidea and the Coleoptera suborder Polyphaga (Hunt et al. 2007). Ladybird beetles are ecologically and morphologically diverse, comprising about 360 genera and nearly 6000 species that range in size from 0.8 mm to 18 mm (Seago et al. 2011). This family also exhibits a broad trophic diversity that encompasses herbivory, pollenophagy, fungivory, and highly specialized predation on coccids (Coccoidea) or aphids (Aphidoidea), but some are predators of aleyrods (Aleyrodoidea), psyllids (Psylloidea), chrysomelids (Chrysomeloidea) or mites (Acari) (Obrycki and Kring 1998; Magro et al. 2010). *Cheilomenes sexmaculata* (Fabricius, 1781), a medium sized ladybird in Coccinellinae, having four orange transverse spots within its elytrum. *C. sexmaculata* is a common natural enemy in agricultural systems which can prey on aphid and psyllid, especially for *Diaphorina citri*, a vector of the destructive pathogen disease of citrus (huanglongbing, HLB). Mitogenome can be utilized in research on taxonomic resolution, population genetic structure, phylogeography and phylogeny. For further study on population genetic structure of *C. sexmaculata*, we sequenced the complete mitogenome of *C. sexmaculata* and analyzed the phylogenetic relationships of Coccinellidae based on mitogenome data.

Male adults of *C. sexmaculata* were collected from Ganzhou City, Jiangxi Province, China (25°47′N, 114°52′E, June 2019) and were stored deposited in the Entomological Museum of Gannan Normal University, Ganzhou, China (please contact Dr. Chanyong Song, email: scyong1229@126.com) under the voucher number GNU-ECQ03. Total genomic DNA was extracted from muscle tissues of the thorax using DNeasy DNA Extraction kit (Qiagen, Hilden, Germany). A pair-end sequence library was constructed and sequenced using Illumina HiSeq 2500 platform (Illumina, San Diego, CA), with 150 bp pair-end sequencing method. A total of 19.8 million reads were generated and had been deposited in the NCBI Sequence Read Archive (SRA) with accession number SRR14150621. With the mitochondrial genome of *Halyzia sedecimguttata* (KT780652) employed as reference, raw reads were assembled using MITObim v 1.7 (Hahn et al. 2013). By comparison with the homologous sequences of other Coccinellidae species from GenBank, the mitogenome of *C. sexmaculata* was annotated using software GENEIOUS R11 (Biomatters Ltd., Auckland, New Zealand).

The complete mitogenome of *C. sexmaculata* is 17,297 bp in length (GenBank accession no. MW845811), and contains the typical set of 13 protein-coding, two rRNA and 22 tRNA genes, and one non-coding AT-rich region. Gene order was conserved and identical to most other previously sequenced Coccinellidae (Nattier and Salazar 2019; Seo et al. 2019 Sheffield et al., 2008; Magro et al. 2020; Song et al. 2020). The nucleotide composition of the mitogenome is 78.0% A + T content (A 40.8%, T 37.2%, C 13.1%, G 8.9%). Four PCGs (*nad1*, *nad4*, *nad4l* and *nad5*) were encoded by the minority strand (N-strand) while the other nine were located on the majority strand (J-strand). All PCGs of *C. sexmaculata* have the conventional start codon for invertebrate mitochondrial PCGs (ATN), with the exception of *cox1* (AAT) and *nad3* (TTG), as the asparagine (AAT or AAC) are proposed to be the start codon for *cox1* in suborder Polyphaga (Sheffield et al. 2008). Except for seven genes (*cox1*, *cox2*, *cox3*, *nad3*, *nad5*, *nad4* and *nad6*) end with the incomplete stop codon T−, all other PCGs terminated with the stop codon TAA or TAG. The 22 tRNA genes vary from 55 bp (*trnS1*) to 70 bp (*trnK*). Two rRNA genes (*rrnL* and *rrnS*) locate at *trnL1*/*trnV* and *trnV*/control region, respectively. The lengths of *rrnL* and *rrnS* in *C. sexmaculata* are 1,296 and 740 bp respectively, with AT contents of 82.6% and 81.1%, respectively.

Phylogenetic analysis was performed based on the nucleotide sequences of 13 PCGs from 20 Coleoptera species. Alignments of individual genes were concatenated using SequenceMatrix 1.7.8 (Vaidya et al. 2011). Phylogenetic tree was constructed through raxmlGUI 1.5 (Silvestro and Michalak 2012). Phylogenetic analysis positioned *C. sexmaculata* in a well-supported clade with *Aiolocaria hexaspilota* (Figure 1), indicating genus *Chilomenes* had a close relationship with *Aiolocaria*. The relationships (Sticholotidinae + (Coccinellinae + (Scymninae + Epilachninae))) were supported in Coccinellidae, and Halyziini was paraphyletic to Coccinellini within Coccinellinae. The monophyly of Coccinellini could not be confirmed by this phylogenetic tree. These results provided an important basis for further studies on mitochondrial genome and phylogenetics of Coccinellidae.

**Figure 1.**
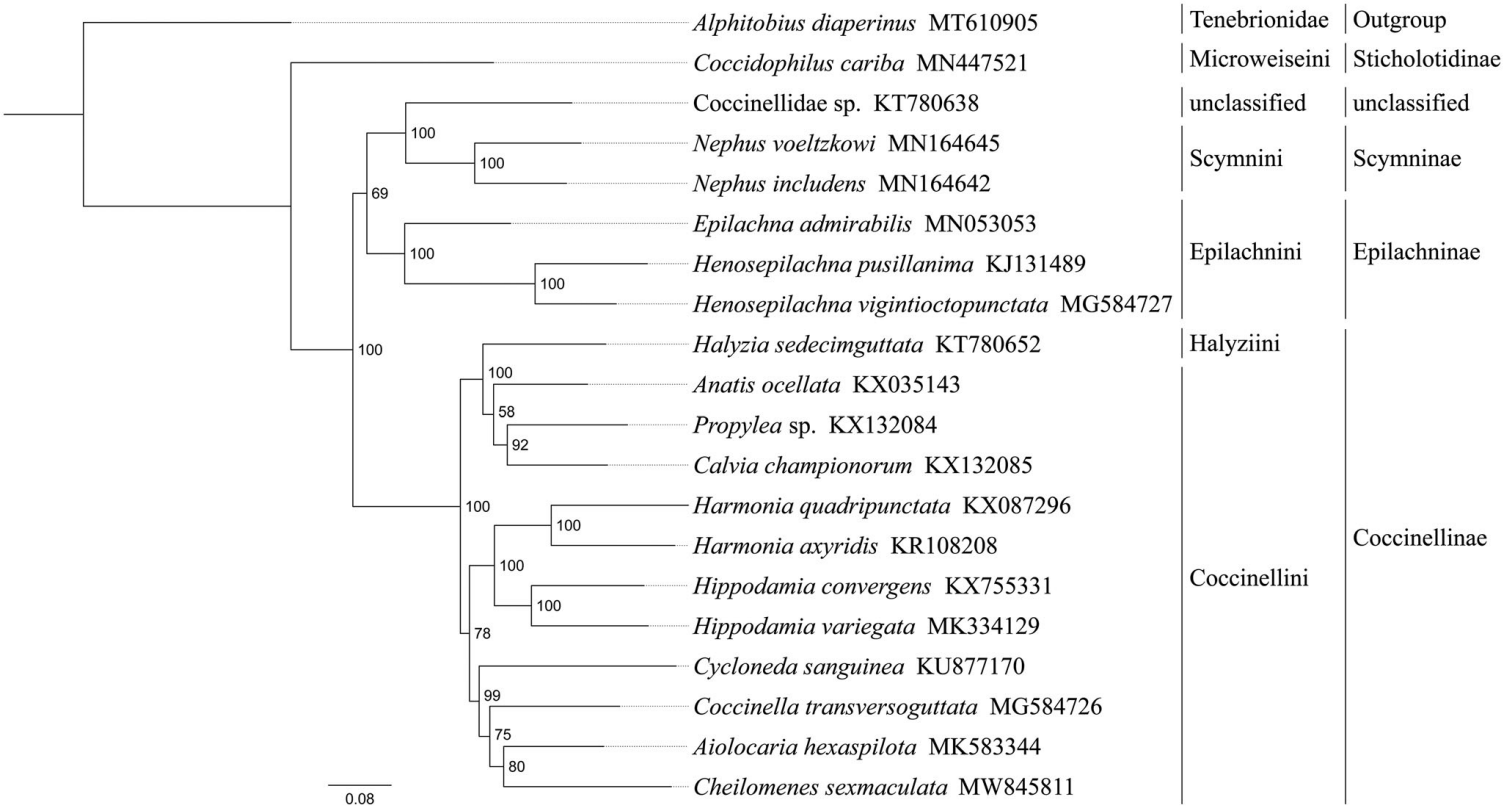
Phylogenetic relationships based on the 13 mitochondrial protein-coding genes sequences inferred from RaxML. Numbers on branches are Bootstrap support values (BS).

## Data Availability

The data that support the findings of this study are openly available in NCBI (National Center for Biotechnology Information) at https://www.ncbi.nlm.nih.gov/, reference number MW845811. The associated BioProject, SRA, and Bio-Sample numbers are PRJNA719170, SRR14150621, and SAMN18590510 respectively.
